# Cilia - the prodigal organelle

**DOI:** 10.1186/2046-2530-1-1

**Published:** 2012-04-25

**Authors:** Phil Beales, Peter K Jackson

**Affiliations:** 1 UCL Institute of Child Health, London WC1N 1EH, UK; 2 Genentech Inc., South San Francisco, CA 94080, USA

## 

Cilia are the oldest known cellular organelle, first described in 1675 by Anthony van Leeuwenhoek in protozoa [1]. He described them as 'incredibly thin feet, or little legs, which were moved very nimbly'. The term 'cilium' (Latin for eyelash) was probably first coined by Otto Muller in 1786 [2]. Structurally and functionally similar to eukaryotic flagella, cilia were originally defined by their motility and for many decades this was their only ascribed purpose. During the latter half of the 19th century came the observation of another class of solitary cilium, which for the most-part was non-motile [3-5]. Zimmerman, who first described 'centralgeissel' (central flagella) in mammalian cells also proposed a sensory role for them, but they received little attention thereafter [5]. The organelle was renamed 'primary cilia' in 1968 [6] because the primary cilium was noted to appear first before multiciliated cells appear in the central nervous system. But their function remained elusive until this past decade. In fact, the revelation that primary cilia have a sensory role, signalling to the cell interior external cues which underlie many human diseases, has somewhat eclipsed research into motile cilia. This split with two cilia categories is however, short lived as more recent evidence indicates that, as long suspected, motile cilia/flagella also have sensory potential (see [7] for a review).

**Figure 1:**
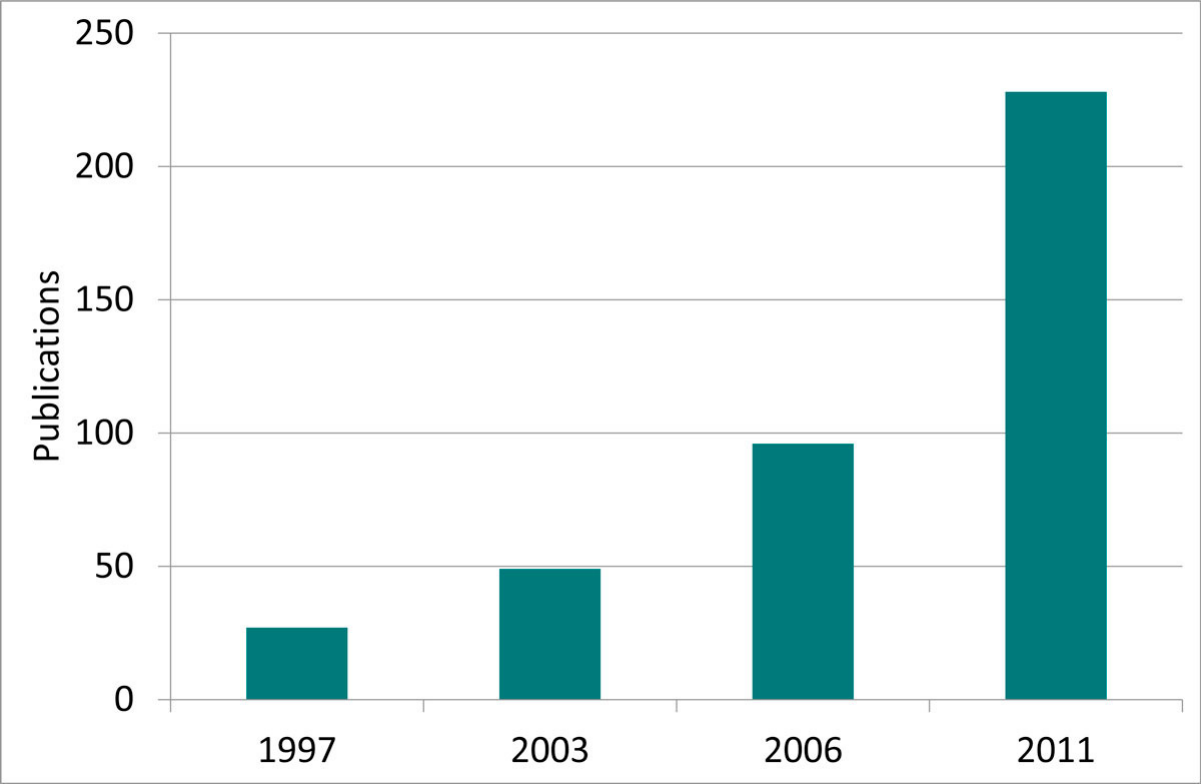
**Publications on primary cilia (Source: PubMed)**.

So why establish a journal devoted to this once forgotten organelle? The reasons are simple: interest and importance. In 1997-1998 there were a handful of publications citing work on primary cilia with the main focus on olfactory receptors (Figure 1). That year however, saw the publication of Nonaka and Hirokawa's seminal paper on nodal cilia and left-right asymmetry which helped kick-start the field [8]. The year 1998 also produced the classic purification of the intraflagellar transport (IFT) complexes from Cole and Rosenbaum [9], providing the molecular basis for previous discovery from Koszminski and Rosenbaum of the intraflagellar transport process [10]. This led in rapid succession to links between polycystic kidney disease and cilia, starting with the link of *C. elegans *homolog of the PKD1 and PKD2 polycystins, mutated in human polycystic kidney disease, to sensory cilia [11]; the link of IFT-B components to mutations in left-right asymmetry [12]; and the link between the IFT-B complex, the polycystic kidney disease gene tg737 and ciliary assembly [13]. In 2003, work from Kathryn Anderson's lab made the striking connection between primary cilia and Hedgehog signaling [14], which caught the attention of developmental biologists and helped bring cilia into the mainstream of developmental cell biology. A variety of links between cilia and morphogen pathways have since been published, causing both enthusiasm and controversy. The year 2006 saw a 20-fold increase in publications on the topic with an emphasis on the role of cilia in polycystic kidney disease. Since 2007, a growing number of publications have characterized interacting networks of proteins that form critical parts of the ciliary trafficking and signaling machinery, and linked these networks and complexes to the human genetic disease, providing deeper explanations for human genetic diseases like Bardet-Biedl syndrome, nephronophthisis, Joubert, and Meckel-Gruber syndromes. Publications in 2011 continued to report a plethora of inherited diseases linked to cilia dysfunction and mechanisms governing trafficking to and within the cilium. Within little over a decade, a new field of biomedical research was born out of observations of this forgotten organelle. This interest will continue unabated for the foreseeable future as new and exciting cellular, developmental and disease-related revelations are made.

It has emerged that the cilium should not be viewed in isolation but rather as intrinsically linked to other organelles - the basal body, centrosome, actin, and microtubular cytoskeleton; to other cellular processes - cell cycle, division, and cytokinesis; and to other signaling pathways important for development: Hedgehog, Wnt, Notch. We are mindful of the need to maintain flexibility and breadth in the types of manuscripts that will be considered for publication in *Cilia*. As reflected in the launch edition, we welcome papers covering the function of the centrosome, as well as aspects of the cytoskeleton of interest to cilia biologists. We are especially interested in articles that help explain links between cilia and disease process. There is a great opportunity to not only connect cilia to human genetic diseases ('ciliopathies'), but to explain the role of ciliary signaling in normal physiology and potentially to link cilia to disease pathologies that are not clearly genetic in nature. To date, we know little about the role that cilia may play in metabolic disease, infectious disease, and only a glimmer of what the role of cilia in cancer may be.

*Cilia *expects to publish a wide range of topics from the structure of cilia to human genetics to ciliotherapeutics and our expectation is that the journal's structure will evolve. This should not be a problem for an online journal. We expect to publish both full research articles and shorter reports. These reports might range from a collection of clinical observations on a ciliary disease, to a human genetics analysis of mutations in a disease cluster, to an 'omics' analysis of some ciliary regulators, and to detailed microscopy revealing a new structure. The reports need not be long, even one figure could be considered. They only need be of immediate interest to our community of cilia biologists, and to have data of high quality. We also welcome solicitations for reviews or opinion pieces. We are committed to a rapid and fair review of papers.

Of paramount importance to both of us as editors is the fact that *Cilia *is an open access title and we are grateful to BioMed Central for their commitment to support its launch and maintain its profile. We have been delighted by the enthusiastic response of the contributing authors whose works are showcased in this first edition.
